# Giant uterine tumor and miscarriage: how to proceed?

**DOI:** 10.1590/1414-431X2024e13549

**Published:** 2024-05-03

**Authors:** E.A. Hase, L.L. Iervolino, H.A. Antico, N. Bozzini, R.P.V. Francisco

**Affiliations:** 1 Departamento de Obstetrícia e Ginecologia, Hospital das Clínicas, Faculdade de Medicina, Universidade de São Paulo, São Paulo, SP, Brasil

**Keywords:** Uterine mass, Giant tumor, Miscarriage, Ultrasound, Myoma

## Abstract

Expanding uterine masses can be the cause of pregnancy loss and add technical difficulties to uterus evacuation due to the intense anatomical distortion of the endocervical canal and uterine cavity. The literature is scarce in the peculiarities of the management of missed abortions in uterus with important distorted anatomies. We report a case of a primigravida patient who presented a rapid and expressive increase of abdominal volume due to a giant uterine mass, evolving to miscarriage. Ultrasound can be a useful tool, allowing visualization of the endocervical path and uterine cavity, helping to perform uterine evacuation in the presence of anatomical distortion without compromising the reproductive future. To the best of our knowledge, no such case has been previously reported.

## Introduction

Uterine myomatosis is frequent in women at menarche and is present even during pregnancy. Fibroids are benign gynecological tumors present in 3-11% of pregnancies (1). In 50-60% of the cases, fibroids remain stable during the gestational period, in 22-32% they increase in size, and in 8-27% they decrease. Growth usually occurs early in the first trimester, preferably from previous fibroids larger than 5 cm and rarely exceeds 25% of its pre-pregnancy volume (2-4). The majority of fibroids remain asymptomatic during pregnancy. When symptomatic, pain is the most common complaint followed by pelvic pressure and genital bleeding. Antepartum surgical treatment should be avoided, and conservative analgesic management is preferred (5,6).

Myomatosis can increase the risk of obstetric complications such as early miscarriage, preterm labor, abnormal fetal presentation, and placental abruption (2,5). Therapeutic options for patients with early pregnancy loss include expectant, medical, and surgical management (7). Expanding uterine masses with intense anatomical distortion of the endocervical canal and uterine cavity can be the cause of pregnancy loss and add technical difficulties to uterus evacuation.

The literature explores poorly the peculiarities of the management of missed abortions in uteruses with very distorted anatomies. Only a few case reports and case series are described due to the rarity of this condition (8,9). In these cases, drug treatment is recommended until complete elimination of the uterine contents because of technical difficulties of conventional surgical evacuation, even though this may take some time, leading to clinical complications in the mother such as pelvic pain, anemia, and risk of infection. Manual vacuum aspiration of the uterus is an alternative method to medical or surgical evacuation in the management of first trimester or incomplete miscarriages, but we did not find its use for distorted uterine cavity in the literature (10). Therefore, we present a case to illustrate our experience with the difficulties of managing a patient with missed abortion in a uterus with an obstructive tumor, and to show how we can proceed to perform uterine emptying in the presence of an anatomical distortion without compromising the reproductive future of the patient.

## Case presentation

Our patient was a 27-year-old woman, primigravida. She was attended at the emergency room of the Hospital Universitário of the University of São Paulo (HU-USP, Brazil) on May 27, 2021 due to an increase in abdominal volume in the previous two weeks associated with menstrual delay. Ultrasound (US) was performed and showed a single embryo with a heartbeat, gestational age compatible with 5 weeks and 6 days, and a heterogeneous abdominal mass suggestive of uterine myomatosis. On June 14, at 8 weeks and 2 days of gestation, the patient presented an episode of genital bleeding associated with abdominal pain of low intensity and went to the emergency service for evaluation. US was performed, which diagnosed a non-evolutive pregnancy. The patient opted for a conservative approach to spontaneously terminate the pregnancy.

**Figure 1:**
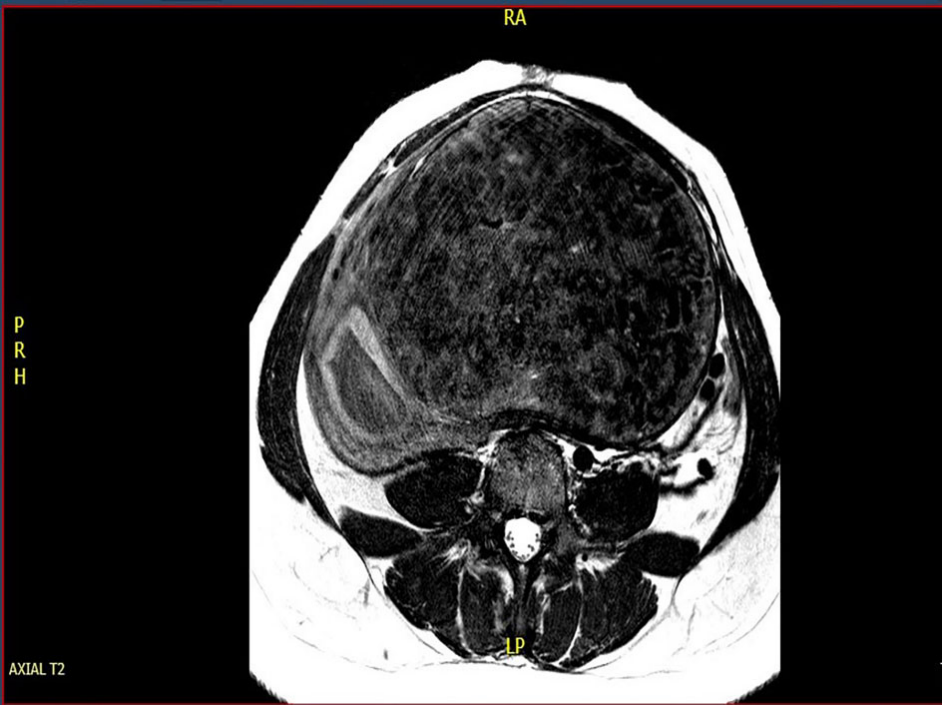
Transverse abdominal section on T2 magnetic resonance imaging showing right cervical deviation due to the mass growth.

On June 17, the patient went to the emergency room at the Hospital das Clínicas, with persistent bleeding and continuous increase of the abdominal volume, without pain or elimination of conceptual products, and she was hospitalized for investigation. MRI was performed on June 18 (Figure 1), which showed an enlarged uterus with an upper limit above the umbilical scar and measuring 22.8×11.8×19.0 cm (volume=2600 cc) and the presence of a voluminous transmural leiomyoma with areas of low signal on T2 images and predominantly hypo-vascular in relation to the myometrium, located on the posterior wall and measuring 18.2×15.8×10.0 cm. The uterine cavity was displaced by the leiomyoma and distended by hematic content (assessment was limited by artifacts) reaching a thickness of 3.8 cm. The significant deviation of the cervical canal due to the effect of the mass could make an active surgical approach difficult. Expectant management was therefore adopted to wait for spontaneous elimination of the abortion (until June 22, 2021).

However, because there was no elimination, after the patient's consent, she received 400 mcg misoprostol vaginally. The cervix was dilated by 1 to 2 cm four hours later and was still shifted to the right due to the obstructing tumor, palpated in the left cul-de-sac. We decided to use manual vacuum aspiration (MVA) guided by US to visualize the endocervical path and uterine cavity. The anterior lip of the cervix was clamped with Pozzi tweezers, and the vacuum cannula was introduced with simultaneous visualization of the endocervical path and uterine cavity by abdominal US. A small amount of material was obtained for anatomopathological (AP) analysis. She was discharged the day after (June 23) without clinical or obstetric complications for the gynecological follow-up of myometrial mass and AP results.

**Figure 2:**
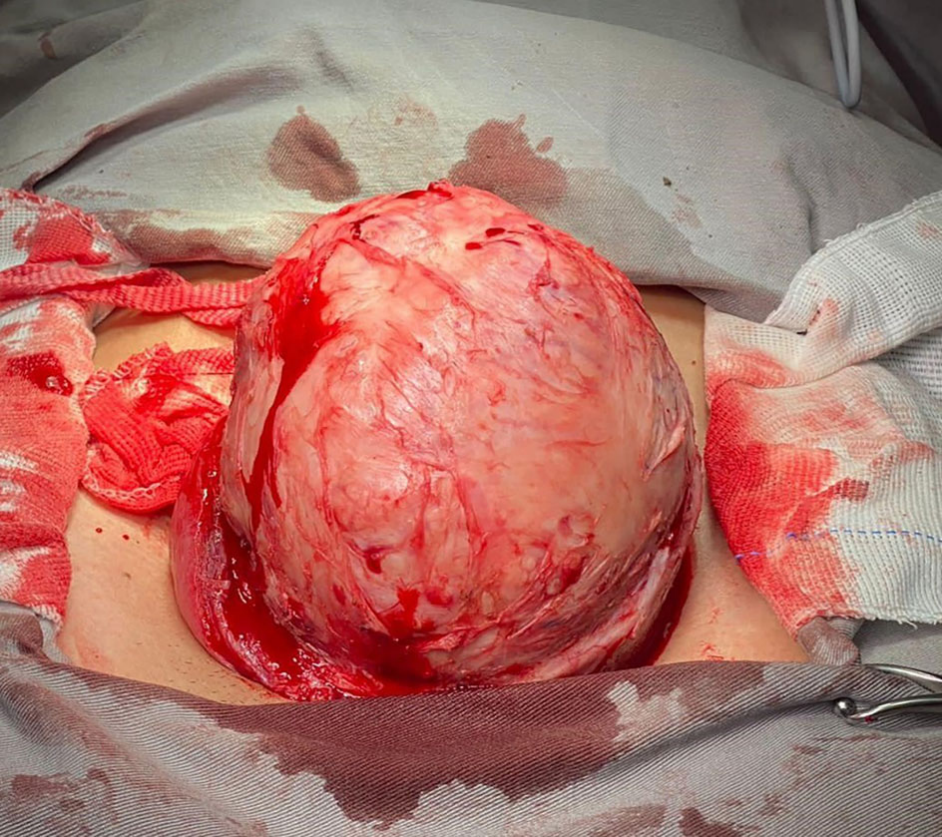
Myometrial mass exposed after uterine wall incision and dissection measuring 14 cm at the largest diameter.

**Figure 3:**
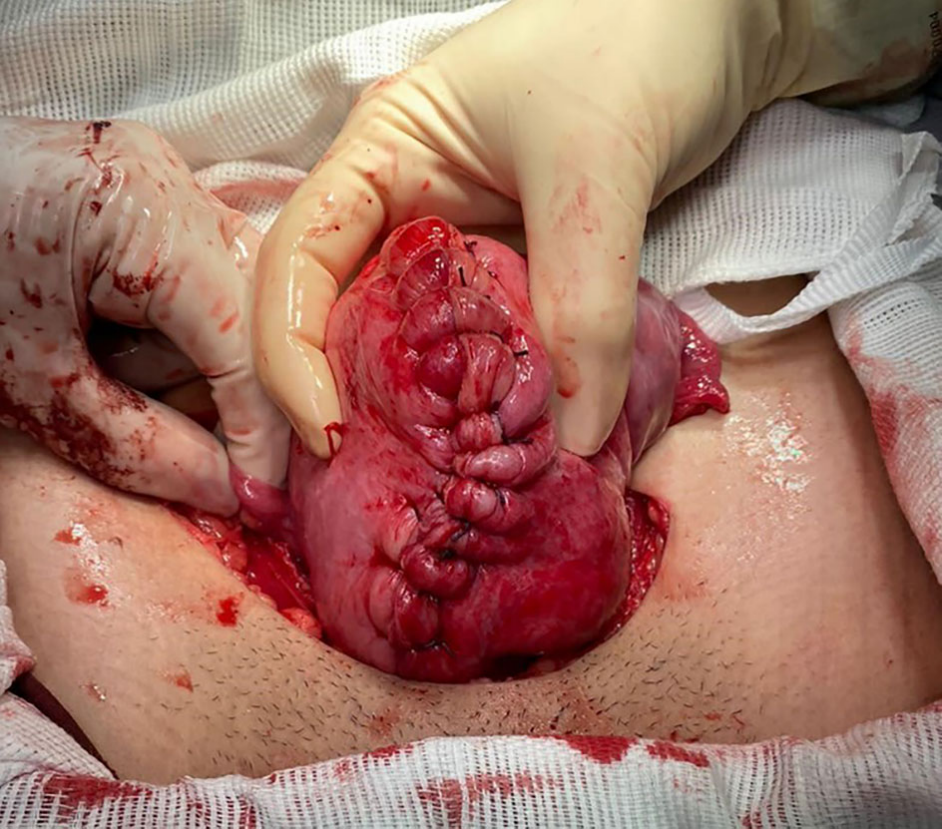
The uterus was sutured after fibroid excision.

The patient attended the gynecology ambulatory clinic on June 30, 2021 and a surgical approach was indicated. Leuprorelin (11.25 mg) was applied on the same day to reduce tumor volume. Laparotomic myomectomy was performed on September 9, 2021, with a vertical myometrium incision with the opening of the cavity and removal of a single myometrial mass measuring about 14 cm at the largest diameter (Figures 2 and 3), without complications. The material was sent for AP analysis. The patient did well in the postoperative period and was discharged after three days. The AP analysis showed a surgical specimen weighing 772 g and histological pattern compatible with uterine leiomyoma with extensive areas of hyalinization suggestive of chronic ischemia and absence of atypia. Hysterosalpingography and hysteroscopy was performed for evaluation of the endometrial cavity, which showed no alteration of uterine cavity and ostia of uterine tubes. A minimum interval of 4 months was recommended before attempting a new pregnancy.

## Discussion

A correct embryo implantation and placentation are necessary for a successful gestation. It is known that in the second week of gestation after ovulation, the trophoblastic invasion hits the wall of uterine spiral arteries, a process that causes modifications on the maternal vasculature and has a fundamental importance to establish an adequate uteroplacental blood flow (11). However, in the myomatous uterus, especially in those with a submucosal component, the decidualized endometrium may appear compressed, presenting a decidual atrophy or distortion of local vascularization, affecting the placentation process and blood supply, impairing embryo development (12). Another theory used to explain the early fetal loses in women with an important tumor growth is an increased uterine contractility or a modification in the production of catalyst enzymes by the placenta, both leading to an (anatomical or functional) inappropriate placentation, culminating in an early abortion (5,12).

In this patient, an expressive increase in uterine volume was noted exclusively during the pregnancy, which may have contributed for its non-evolution. The growth pattern observed was compatible with those described in the literature, which describes a higher possibility of myomatous growth during the first trimester compared to the third (3). Although the exact pathophysiology of this growth is not fully understood, it is known that sex steroids have an influence. It is believed that human chorionic gonadotropin (HCG) may play an important role in stimulating development via the LH/HCG receptor complex present in the uterine fibroids. This explains the greater frequency during the first trimester of pregnancy since these receptors decrease with pregnancy progression (3,13).

One of the concerns of the assistance team when faced with a fast-growing uterine mass is the neoplastic differential diagnosis: uterine sarcoma, uterine carcinosarcoma, or metastasis can be causes of uterine mass. The final diagnosis is given after a histopathological analysis through the mitotic index, presence of cellular atypia, cellularity, and areas of necrosis (14). In this case, the sarcoma hypothesis was made at first, but radiologic exams were suggestive of leiomyoma, which was confirmed by the histological study of the surgical specimen.

Another concern is to define the best treatment for the patient. In those with infertility caused by the uterine fibroid, the surgical approach with a preconceptionally myomectomy can increase the chances of success, since it decreases uterine contractility and local inflammation. There is no significant difference between the laparotomy or laparoscopy approach in subsequent gestational success or the risk of uterine rupture during pregnancy (estimated at 0.2 and 0.26%, respectively). On the other hand, during gestation, myomectomy is not the first choice of treatment. It can be considered in specific situations such as uncontrollable pain, sprains in pedunculated fibroids, and fast-growing tumors that cause compression of other organs. If myomectomy is necessary, it should preferably be performed after the first trimester, especially after the puerperium. A myomectomy during the cesarean section is not recommended, but it can be performed in selected situations like pedunculated subserosal fibroids (15).

As already pointed out, despite the frequent description of uterine fibroids during gestation in the literature, peculiarities of the management of missed abortions in these uteruses with severely distorted anatomy are poorly researched because of the rarity of this condition, and only a few case reports and case series are described (9). Possible options include expectancy, medical approach with or without uterine curettage, or surgical approach with myomectomy. Mark et al. (8) described a case series (n=12) of abortions in uteri with voluminous fibroids. Patients were submitted to a single dose of 200 mg mifepristone orally, followed by 800 mcg misoprostol vaginally 24 and 48 h later for those under 14 weeks of gestational age, and patients beyond 14 weeks received 400 mg misoprostol vaginally every 4-6 h until delivery. The medical approach was successful in 8 patients and 4 needed uterine curettage. The proposed regimen was considered effective and safe, with a low complication rate (8). Santibenchakul and Jaisamrarn (9) used a combination of drugs for medical abortion in a first-trimester pregnancy with large multiple uterine myomas, but two weeks after the initial regimen an additional dose of misoprostol vaginally was required for the complete uterine evacuation. An alternative method for the management of first trimester or incomplete miscarriages for the medical or surgical evacuation is the MVA of the uterus, but its use is not described for a distorted uterine cavity (7).

In this present case report, because there was a severe cervical canal deviation caused by the mass and the patient's reproductive desire, the expectant approach was firstly chosen for the spontaneous elimination of abortion. As it was not successful, a medical approach followed by uterine aspiration guided by ultrasound was made. The ultrasound used in this procedure provided greater safety and efficacy to the surgical approach. This case illustrates the importance of therapeutic planning when distortions are present in the uterine cavity and we recommend using ultrasound as a guide for uterus emptying to avoid the risk of complications such as incomplete emptying, uterine perforation, hemorrhage, cervical laceration, false passage, and visceral injuries. This is a strength of our case report, as there are no guidelines for the type of intervention in these cases. To the best of our knowledge, no such case has been reported in the literature, and therefore, our outcomes cannot be compared to other studies. A myomectomy performed posteriorly, without surgical or clinical complications, benefits the reproductive life of the patient.

Expanding uterine masses can be the cause of pregnancy loss and add technical difficulties to uterus evacuation due to severe distortions of the endocervical canal and uterine cavity. Ultrasound, a non-invasive imaging method, can be useful in these cases as it allows visualization of these structures and thereby helps in performing the procedure by serving as a guide and providing greater safety, easiness, and efficacy to patient approach.
